# High-throughput Optical Analysis to Inform Design of Electrochemical Biosensors

**DOI:** 10.1021/acsmeasuresciau.6c00018

**Published:** 2026-03-30

**Authors:** Nathan J. Ricks, Michael A. Pence, Monica Brachi, Shelley D. Minteer

**Affiliations:** Kummer Institute Center for Resource Sustainability, 14717 Missouri University of Science and Technology, Rolla, Missouri 65409, United States

**Keywords:** high-throughput screening, biosensors, electrochemistry, enzyme engineering, fluorescent spectroscopy

## Abstract

Electrochemical biosensors are central to wearable diagnostics, point-of-care testing, and continuous health monitoring due to their low power requirements, compatibility with miniaturized electronics, and proven clinical impact. Despite these advantages, the development of new electrochemical biosensors remains slow, constrained by limited throughput, complex electrode–biomolecule interfaces, and challenges associated with selectivity and performance in chemically complex environments. This perspective outlines how the next generation of electrochemical biosensors can be enabled by decoupling high-throughput front-end discovery and optimization from electrochemical readouts using nonelectrochemical surrogate assays. Optical, affinity, and cell-sorting platforms, including SELEX, fluorescence-activated cell sorting, and chemically coupled fluorescence assays, allow orders-of-magnitude expansion in accessible design space for recognition elements, enzymes, and redox mediators. These approaches enable data-rich exploration of sequence–function relationships and provide scalable inputs for directed evolution, de novo protein design, and machine-learning-guided optimization. Top-performing constructs obtained from these nonelectrochemical surrogate assays can then be screened and validated electrochemically, ensuring translation into functional electrochemical biosensors. Together, these strategies outline a path toward data-driven, scalable, and predictive electrochemical biosensor design that moves beyond trial-and-error development and accelerates deployment in real-world settings.

## Graphical abstract

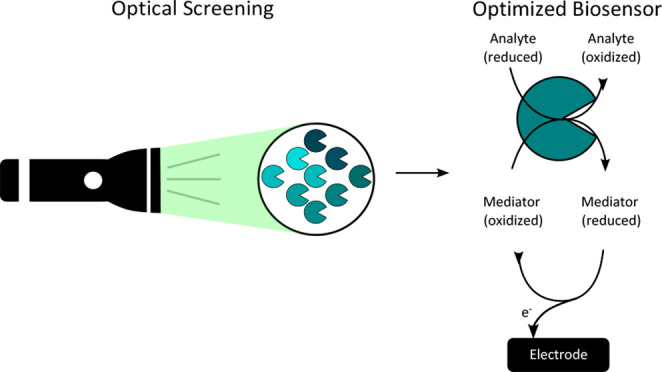

## 1 Introduction

As medicine, environmental monitoring, drug screening, and personalized health technologies continue toward real-time, decentralized, and data-rich sensing, the demand for precise, selective, and scalable measurement of specific chemical and biological species has grown dramatically.^1–5^ Modern diagnostics increasingly rely on detecting low-abundance metabolites, proteins, and signaling molecules in complex, dynamic environments, often outside of traditional laboratory settings. Meeting these demands requires analytical platforms that are not only sensitive and selective but also robust, miniaturizable, and compatible with continuous or point-of-care operation.

Biosensors address this challenge by coupling a biological or biomimetic recognition element to a physical transducer, enabling molecular recognition events to be converted into quantifiable signals.^6,7^ Depending on the mode of transduction, biosensors can report analyte binding or turnover through optical, mass-sensitive, or electrochemical outputs (Figure 1).^8^ Optical biosensors, such as fluorescent or luminescent reporters, have been invaluable in laboratory and imaging contexts, offering high sensitivity and spatial resolution.^7,9^ However, their reliance on external optics, susceptibility to photobleaching, and limited capability to work outside of a laboratory setting can constrain their deployment in portable or implantable systems.^10–13^

**Figure 1:**
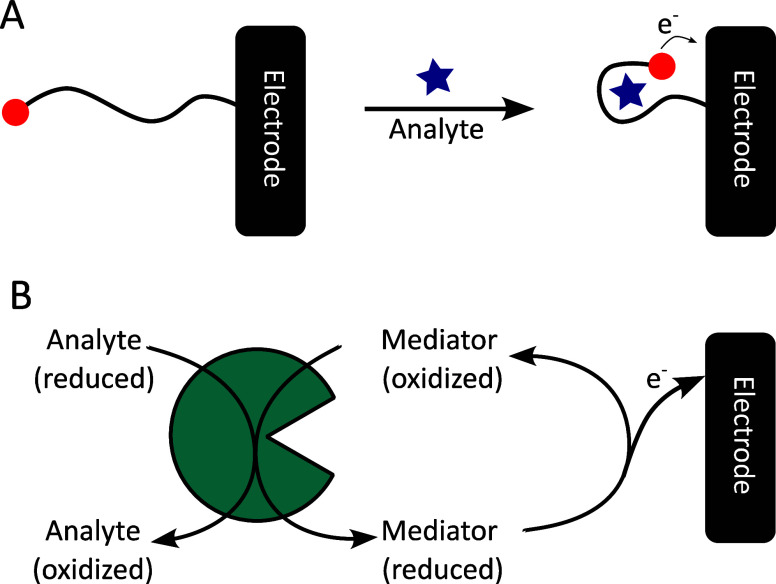
Two classes of electrochemical biosensors: (A) structure-switching sensor and (B) catalytic sensor.

In contrast, electrochemical biosensors directly translate biochemical events into electrical signals. This mode of transduction is particularly attractive due to its intrinsic compatibility with miniaturized electronics, low power requirements, and straightforward integration into wearable, implantable, or sensing platforms.^8,14^ Since the first electrochemical glucose sensor was introduced by Clark in 1962, electrochemical biosensing has expanded beyond single-analyte measurements driven by advances in electrode materials, surface chemistry, and microfabrication technologies.^15,16^

Electrochemical biosensors can be broadly categorized into two dominant mechanistic classes based on how molecular recognition is transduced into an electrical signal. In structure-switching (affinity-based) sensors, analyte binding induces a structural or positional change in the recognition element that alters electron transfer between an electroactive reporter and the electrode surface (Figure 1A). These sensors typically rely on aptamers, antibodies, or engineered nucleic acid constructs and offer reversible, nonconsumptive detection.^17–20^ In contrast, catalytic (activity-based) sensors generate a signal through enzymatic turnover of the target analyte, producing or consuming electrons either directly or via redox mediators (Figure 1B). This class includes widely used enzyme-based sensors and benefits from intrinsic signal amplification through catalysis.^21,22^ Depending on the transduction mode and electroanalytical method used, biosensors can be further differentiated into amperometric, voltammetric, potentiometric, and self-powered. Self-powered sensors are currently of great interest as they can operate galvanostatically, eliminating the need for an external power source. In such systems, electrical power is generated in the presence of analyte and is proportional to analyte concentration. Often, self-powered sensors consist of an analyte-specific oxidoreductase enzyme at the anode coupled with oxygen reduction at the cathode. Microbial and organelle-based devices have also been developed as alternatives to enzymatic devices, taking advantage of more complex biochemical cascades.^23–25^ While other electrochemical formats, such as impedimetric or transistor-based sensors, employ different modes of signal transduction, they generally rely on either affinity-based binding or catalytic activity as the underlying recognition mechanism.

Despite their promising characteristics, the translation of electrochemical biosensors from laboratory demonstration to widespread practical use has remained limited. Currently, FDA-approved devices are largely restricted to glucose monitoring and selected cardiovascular measurements, underscoring persistent challenges related to biorecognition elements availability, selectivity in complex matrices, and device-to-device reproducibility.^26,27^ These challenges are especially pronounced in point-of-care and continuous monitoring applications where sensors must operate reliably across fluctuating physiological and environmental conditions.^28^

Addressing these limitations requires not only advances in materials and device architectures, but also new strategies for sensor discovery, optimization, and adaptation. Historically, the optimization of electrochemical biosensors has been constrained by the inherently low throughput of electrochemical screening methods, limiting systematic exploration of design space. In contrast, high-throughput (HTP) screening approaches are well established in synthetic and chemical biology, where large libraries of biorecognition elements and molecular variants can be evaluated rapidly. Leveraging these nonelectrochemical HTP assays as surrogate screening tools offers a powerful route to accelerate the identification and optimization of electrochemical biosensor components (Figure 2). Such approaches enable rapid exploration of biorecognition elements and design parameters that would be impractical to assess using electrochemical measurements alone. This perspective aims to highlight emerging opportunities to harness nonelectrochemical surrogate assays to guide the design, optimization, and deployment of next-generation electrochemical biosensors.

**Figure 2:**
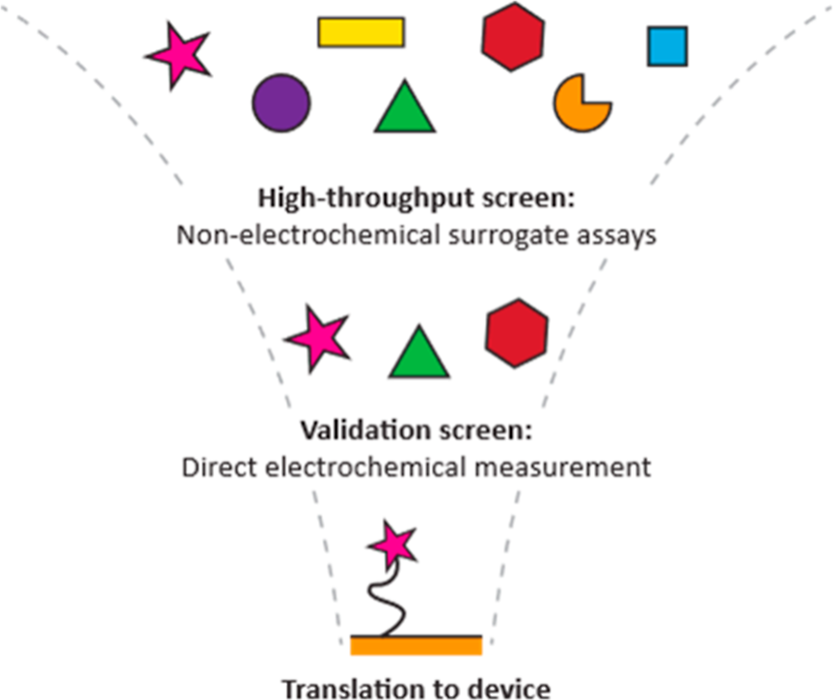
Schematic showing the different tiers of screening protocols for developing electrochemical biosensors.

### 1.1 Challenges and Opportunities for HTP Electrochemistry

#### 1.1.1 Key Technologies and Challenges in HTP Electrochemistry

Incorporating direct electrochemical measurements into discovery-scale screening and selection workflows would benefit from experimental throughput that matches common library screening techniques. There have been commercially successful examples of HTP electrochemical screening such as Roche’s electrochemiluminescence assays (>10^3^ tests/hour), but these assays use an optical signal as a readout which would complicate translation to a portable sensing device.^29^ There is a need for a direct electrochemical characterization platform with this same level of throughput. In recent years, there have been many efforts in increasing the throughput of electrochemical characterization, but many platforms struggle to provide the speed, parallelization, and coupling to biological workflows that is needed to facilitate large scale library screening.^30^ We highlight here pathways to HTP direct electrochemical measurements, which can be used to more efficiently validate the results of nonelectrochemical surrogate assays.

Currently, direct electrochemical measurements suffer from two major throughput bottlenecks: experiments are typically not as parallelized as optical methods and serial experimental workflows are typically slow and inefficient. This can be solved by parallelizing electrochemical measurements and decreasing experimental time of upstream sample and electrode preparation. This is primarily accomplished using multichannel potentiostats, multielectrode arrays, and laboratory automation.^31^ While these technologies are effective in increasing throughput, they are often not commercially available and have yet to be implemented in extremely large-scale exploration of sequence space. However, these technologies will be key to attaining the throughput required for data-driven electrochemical biosensor development. Below, we provide a brief overview of existing technologies that could be coupled with nonelectrochemical assays for rapid validation and translatable development of electrochemical biosensors.

The most compelling method for increasing throughput is to simply run multiple experiments at the same time.^32^ To this end, electrochemical workstations with simultaneous control of multiple electrochemical channels are highly valuable. However, there are limited commercial options that offer the throughput required to integrate with biological screening campaigns. Despite this lack of commercial options, there have been many research groups developing multichannel potentiostats, which can enable parallel and HTP electrochemistry.^33–35^ One recent example of this is Legion (Figure 3A), a potentiostat which has 96 unique channels with independent potential and current control and measurement, allowing for fully parallelized electroanalysis.^36^ The 96-wellplate format makes it compatible with automated biological workflows, allowing for top performers from a library screen to be tested by direct electrochemical measurements.^37^

**Figure 3:**
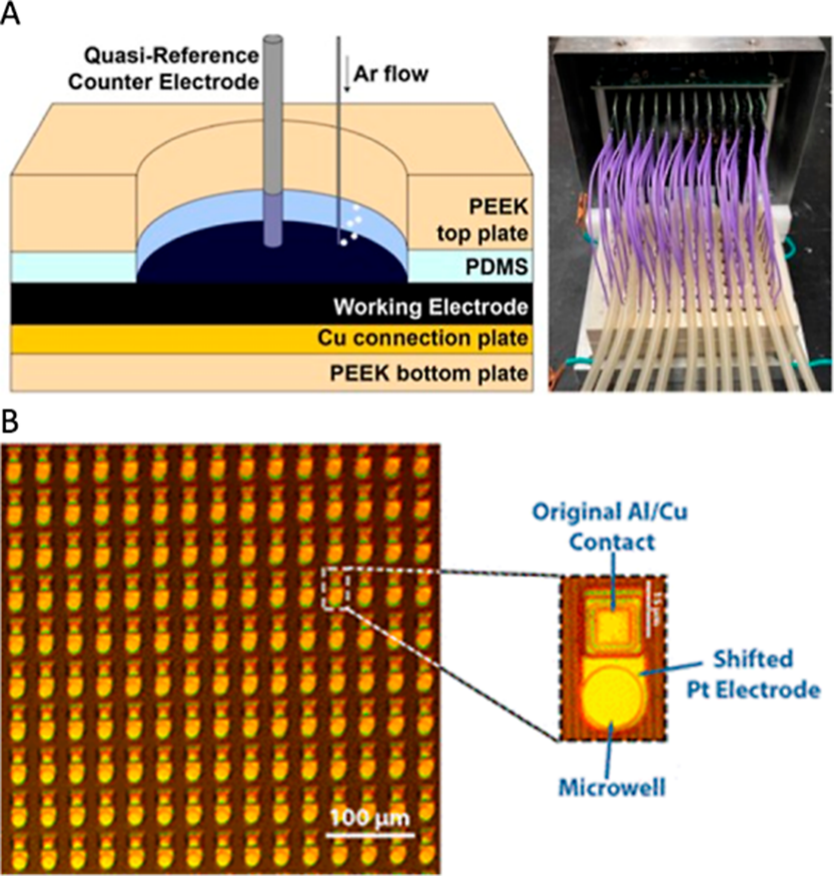
Methods for accelerating electrochemical screening of biological constructs. (A) Legion, a 96-channel potentiostat for obtaining highly parallel electroanalytical data. Adapted with permission under Creative Commons CC-BY–NC–ND 4.0 from ref 36. Copyright 2023 American Chemical Society. (B) A 1024 microwell electrode array. Adapted with permission under Creative Commons CC-BY–NC–ND 4.0 from ref 38. Copyright 2021 American Chemical Society.

Microfabricated multielectrode arrays provide another strategy to increase the experimental throughput of electrochemical characterization. Arrays such as the one shown in Figure 3B can have upward of a thousand individually addressable electrodes, which are often directly integrated with potentiostat circuitry.^38^ Multielectrode arrays are used to screen a variety of electrochemical systems, ranging from organic reactions to materials electrodeposition.^39,40^ However, screening of electrochemical biosensors using these arrays has yet to match the throughput of nonelectrochemical surrogate assays, despite promising initial demonstration.^40^ When combined with multichannel potentiostats, these platforms have the possibility to achieve truly HTP electrochemical screening (>10^3^ samples/hour), but they will need equally HTP electrode modification, as the shared electrolyte environment of all the arrays necessitates electrode modification.^30^ Techniques such as polymer-pin lithography and inkjet printing could provide the multiplexed electrode fabrication that are needed to most effectively apply this technology.^41–43^

Laboratory automation is an emerging tool to increase throughput of direct electrochemical characterization. The use of lab automation in electrochemistry has rapidly increased over the past few years, and a broad overview is discussed in the review by Pence et al.^31^ Notably, laboratory automation increases throughput by enabling passive experimentation, i.e., the removal of human intervention allows for 24/7 continuous experiments and data collection, while also significantly improving the reproducibility of experiments.^44^ We believe that coupling of automated electrochemical characterization with existing workflows in chemical biology will provide a key pipeline to rapid development of new sensing constructs.^45,46^ Despite extensive applications in energy storage, synthesis, and electrocatalysis, automated electrochemical characterization has been relatively underutilized in the field of biosensors. Previous efforts in automated electrochemical characterization of biosensors have focused on studying sensitivity toward a target analyte.^47–49^ Moving forward, we are excited about the use of automated electrochemistry to understand selectivity and cross-reactivity to a broad swath of potential interferents.

Figure 4 highlights our group’s efforts in applying automation to gain holistic understanding of sensor selectivity. In this work, automated electrochemistry was used to validate the performance of a modified aptamer sequence previously generated through a SELEX process (see below).^50,51^ Figure 4A shows the aptamer’s response across a scope of 40 interferents. Parametric modeling was used to identify the computed partition coefficient (MolLogP) as the most impactful feature, as shown in Figure 4B. We found that the aptamer had a lower affinity than when determined by fluorescent assays and had significant cross reactivity that was determined mostly by the hydrophobicity of the interferent. This information is highly valuable and can be used to redesign the selection process to incorporate counter-selection rounds as a means of increasing sensor selectivity, confirming the need for a direct electrochemical validation step in the screening process.^52^

**Figure 4:**
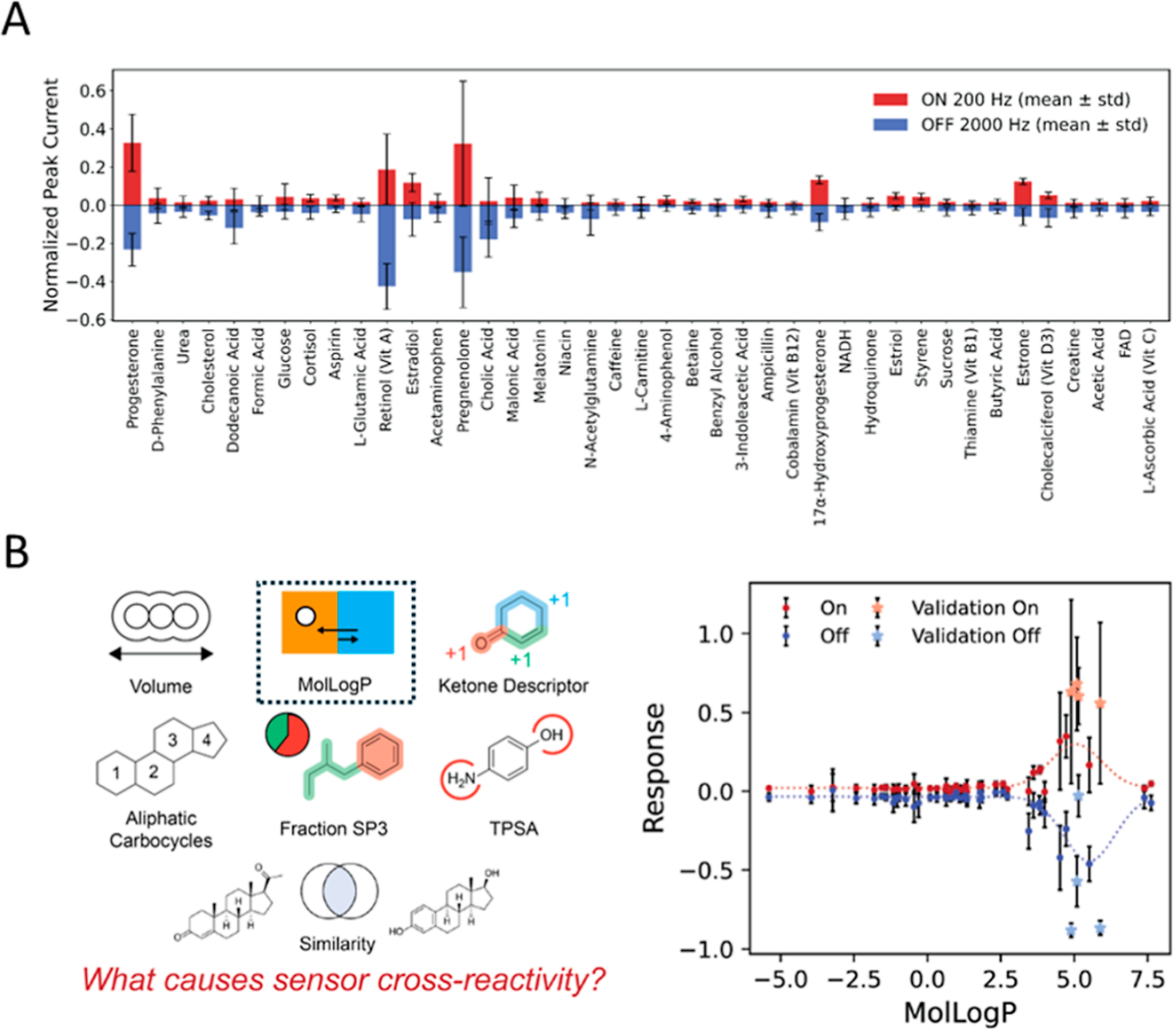
Using automation to better understand aptamer based electrochemical biosensors. (A) Aptamer responses across a broad scope of interferents. (B) Physical descriptors of interferents (left) and the most effect of the most impactful descriptor, MolLogP, on aptamer response, showing that the aptamer responds generally to interferents with a MolLogP of ∼5. Reprinted with permission under a Creative Commons CC BY 4.0 from ref 51. Copyright 2026 ChemRxiv.

Despite the benefits of the above technologies, it is still difficult to achieve electrochemical characterization with the through-put seen in traditional chemical biology techniques. However, we note that electrochemical experiments are of higher-fidelity than nonelectrochemical surrogate assays and are critical to ensure that artificial selection processes are not mislead by the assay.^53^ We envision that a blend of all the above technologies will enable incorporation of nonelectrochemical front-end screening with direct electrochemical measurements as a validation screen. This represents an exciting possibility for truly end-to-end screening processes for the development of electrochemical biosensors.

### 1.2 Nonelectrochemical Assays for High-throughput Screening

#### 1.2.1 DNA Aptamer Screening

Multiple strategies discussed previously in the section above aim to make direct electrochemical characterization a more suitable validation screen, but direct electrochemistry is still poorly suited for front-end large-scale exploration of sequence space. Nonelectrochemical assays such as SELEX and fluorescence-activated cell sorting (FACS) provide a powerful complementary strategy by enabling rapid screening and evolution of biosensor components prior to integration into electrochemical workflows.^19,54–56^ By converting molecular recognition or redox activity into sequence enrichment (SELEX) or sortable optical phenotypes (FACS), these surrogate assays allow for large increases in throughput. Order-of-magnitude increases in throughput allow for directed evolution, combinatorial optimization, and systematic interrogation of design parameters. Importantly, these approaches are useful for early stage discovery and optimization, reserving electrochemical validation for a narrowed set of top-performing candidates. As such, nonelectrochemical HTP assays should represent a foundational front-end measurement layer for electrochemical biosensor development.

Systematic evolution of ligands by exponential enrichment (SELEX) is an example of a nonelectrochemical, HTP screening strategy that enables exploration of molecular recognition space at scales inaccessible to electrochemical measurements and is optimal for aptamer discovery. In a typical SELEX experiment, a diverse oligonucleotide library, often containing 10^13^–10^15^ unique sequences, is synthesized, followed by subsequent testing of binding affinity for the target analyte (Figure 5A). Within this vast sequence space, only a small fraction of molecules adopts folded structures capable of binding the target analyte, which may include proteins, small organic molecules, or ions. During selection, the target is immobilized or otherwise presented, and the oligonucleotide library is exposed under defined conditions; nonbinding sequences are removed, while bound sequences are recovered and amplified by PCR to regenerate an enriched pool. Iterative rounds of binding, partitioning, and amplification are performed, with increasing stringency applied.^20,54,57–59^ Importantly, this process enables the exploration of large sequence spaces that are fundamentally inaccessible to direct electrochemical screening.

**Figure 5:**
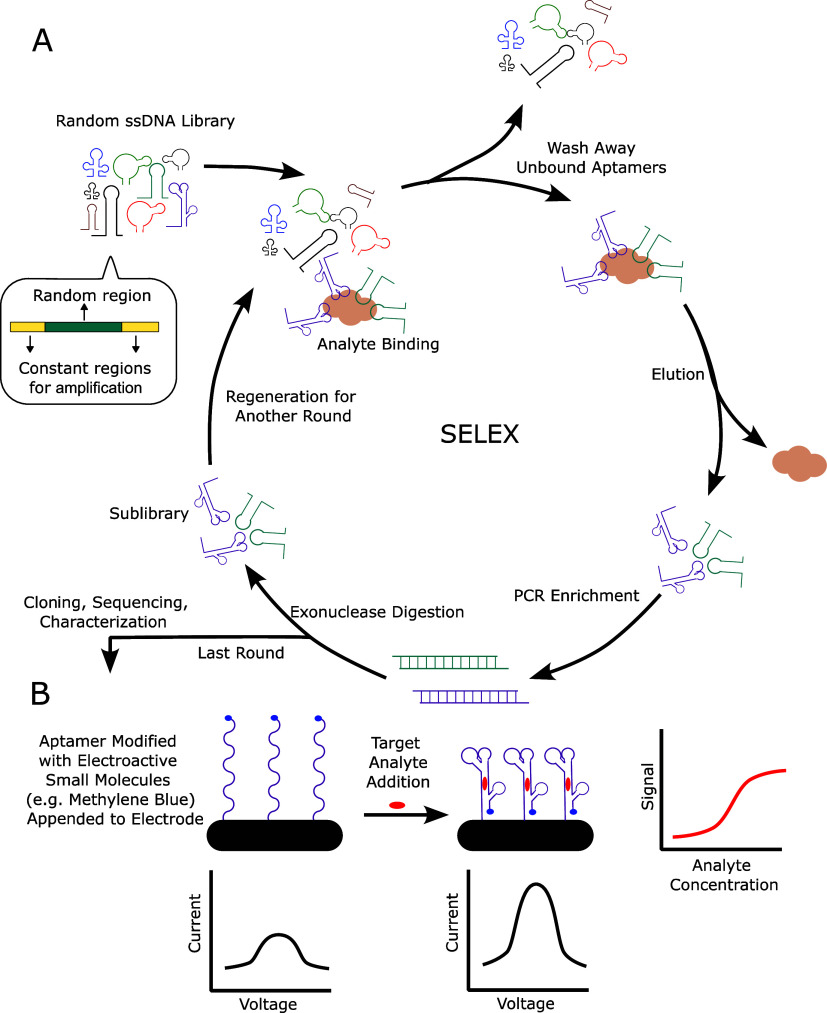
(A) Schematic overview of systematic evolution of ligands by exponential enrichment (SELEX) as a HTP platform for selecting oligonucleotide aptamers from large random libraries through iterative cycles of binding, partitioning, amplification, and enrichment. (B) Application of SELEX-derived aptamers as electrochemical sensors.

For electrochemical biosensor development, SELEX functions as a HTP nonelectrochemical front-end for sensor optimization, enabling molecular recognition to be evolved and refined before electrode integration (Figure 5B). Aptamers isolated through SELEX often undergo ligand-induced conformational changes and can be readily functionalized and immobilized on electrode surfaces, where target binding events are transduced into electrochemical signals such as changes in current, potential, or impedance.^19,20,60^ In many aptamer-based electrochemical sensors, this signal arises from a redox reporter (e.g., methylene blue or ferrocene) appended to the aptamer, for which target-induced conformational rearrangements modulate the distance or orientation of the redox label relative to the electrode surface, thereby altering electron-transfer efficiency.^61–64^ By decoupling recognition element discovery from electrochemical readout, SELEX allows for rapid interrogation of affinity across large candidate pools otherwise intractable by electrochemical techniques, and reduces the experimental burden associated with electrochemical testing. As such, SELEX exemplifies how nonelectrochemical assays can serve as scalable surrogate screening tools that feed directly into data-driven electrochemical biosensor design.

#### 1.2.2 Enzyme Screening by Fluorescence

While SELEX enables HTP optimization of molecular recognition elements for oligonucleotide aptamers, analogous strategies for improving catalytic enzymes used in electrochemical biosensors have progressed more slowly. Directed evolution is a powerful and generalizable approach for enhancing enzyme activity, specificity, and stability, and should have broad applications in improving bioelectrocatalytic sensor systems, yet it has seen limited application in electrochemical biosensor development (Figure 7A).^65–67^ To date, most electrochemical biosensors have been improved through empirical, trial-and-error-driven optimization of naturally occurring systems, including tuning enzyme loading, mediator identity, redox polymer composition, and electrode interfaces, rather than through systematic, large-scale exploration of protein sequence space.^68,69^ In parallel, rational chemical design of redox mediators and polymer architectures has emerged as an alternative strategy to improve bioelectrocatalytic performance without modifying enzyme sequence.^70–74^ Previously, our group has used this rational design strategy to identify redox mediators that can efficiently perform extracellular electron transfer to *Escherichia coli* (Figure 6A).^73^ Similar efforts have been carried to design redox mediators for efficient electron transfer with glucose oxidase (Figure 6B).^74^ Despite efforts in mediator design, the limited application of directed evolution to electrochemical biosensors has likely stemmed from the absence of scalable, HTP electrochemical screening platforms. However, recent advances in chemical biology can provide nonelectrochemical surrogate assays that enable functional evolution at scale using electrochemically relevant proxies as discussed below.

**Figure 6:**
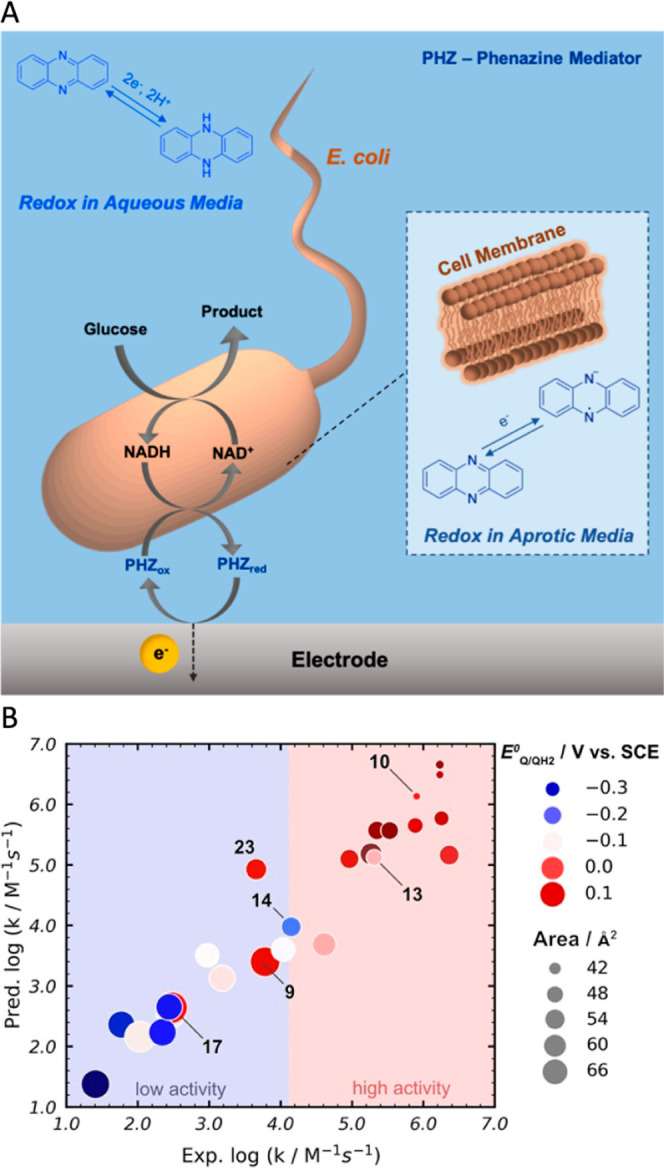
Using structure–function relationships to rationally design (A) phenazine mediators for extracellular electron transfer and (B) quinone mediators for glucose oxidase enzymatic bioelectrocatalysis. (A) Reprinted from iScience, Vol. 24, Rhodes, Z.; Simoska, O.; Dantanarayana, A.; Stevenson, K. J.; Minteer, S. D., using structure–function relationships to understand the Mechanism of Phenazine-Mediated Extracellular Electron Transfer in *Escherichia coli*, 103033, Copyright (2021), with permission from Elsevier. (B) Reproduced with permision from ref 74. Copyright 2023 American Chemical Society.

FACS is a widely used technique that enables individual cells to be rapidly analyzed and physically sorted based on fluorescence intensity, allowing millions of variants to be screened per hour (Figure 7D).^75,76^ Recent FACS-based platforms demonstrate how redox chemistry can be repurposed into HTP, nonelectrochemical selection schemes. In one such approach, glucose oxidase enzymes displayed on the surface of individual cells generate hydrogen peroxide as a reaction product, which in turn initiates the formation of a fluorescent hydrogel localized around the cell surface. Because hydrogel formation is spatially confined to catalytically active cells, fluorescence intensity directly reports on enzymatic turnover, enabling millions of variants to be screened and enriched in a single experiment (Figure 7B).^55,56^ These strategies have been used to evolve oxidoreductases by coupling hydrogen peroxide production to fluorogenic hydrogel formation, allowing rapid enrichment of high-activity variants, which can then be validated by direct electrochemical measurements.

**Figure 7:**
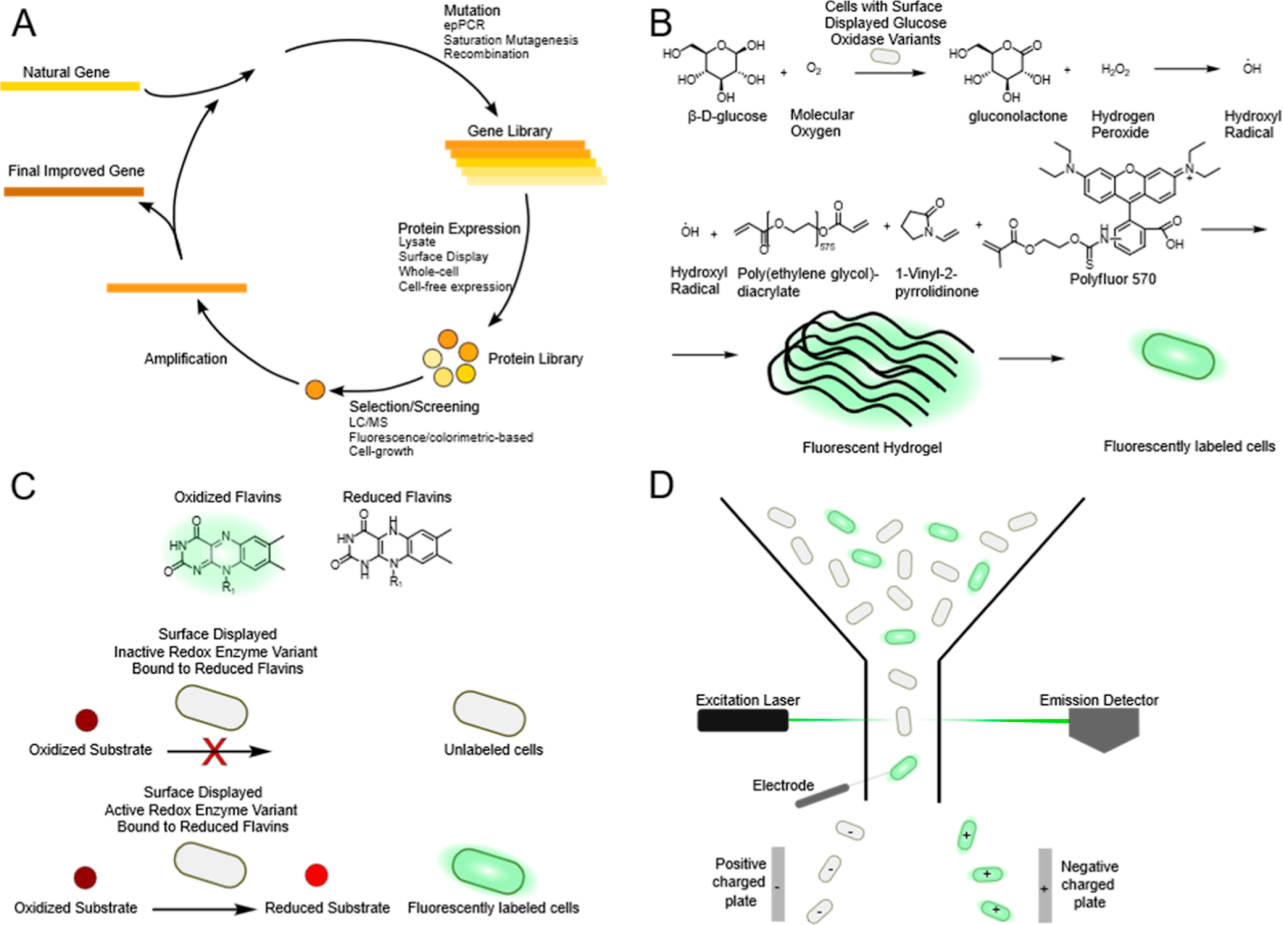
(A) Directed evolution overview for improving protein function through mutation and selection. (B) An example application of directed evolution to improve glucose oxidase activity by coupling enzymatic function to the formation of a fluorescent hydrogel surrounding individual cells. (C) Proposed surrogate screening strategy for redox enzymes that utilize cofactors with oxidation-state–dependent fluorescence, enabling optical readout of redox activity outside of an electrochemical context.(D) Overview of fluorescence-activated cell sorting (FACS) as a HTP platform for linking optical phenotypes to genotype during enzyme and biosensor component optimization.

Beyond hydrogen peroxide-forming enzymes like glucose oxidase, FACS-based screening strategies should be extensible to other redox-active enzymes. We propose that enzymatic activity need not be reported through product accumulation, but through changes in the oxidation state of the cofactor whose optical properties are redox dependent. Any enzyme that catalyzes oxidation–reduction reactions involving cofactors such as flavins, NAD­(P)­H, phenazines, or pterins can, in principle, be screened using analogous fluorescence-based proxies (Figure 7C).^77–80^ By translating catalytic redox cycling into an optical phenotype, such FACS-based screening approaches enable rapid, HTP evolution of redox enzymes beyond those that generate hydrogen peroxide as a byproduct.

By converting redox turnover into a sortable fluorescence phenotype, FACS-based assays provide a scalable route to optimize sensing enzymes, redox mediators, and electron-transfer pathways prior to electrode integration. As with SELEX, these approaches decouple HTP functional screening from electrochemical readout, positioning FACS as a powerful assay for electrochemical biosensor design and enabling directed evolution of electrochemical biosensors. Even beyond fluorescence-based screening, many redox reactions can be coupled to optical outputs. Notably, directed evolution of multicopper oxidases such as laccases has successfully employed simple colorimetric assays to screen large mutant libraries for improved redox activity and substrate turnover, demonstrating the scalability and sensitivity of optical surrogates for redox catalysis.^81,82^ Together, these examples underscore the breadth of readouts for selection, including fluorescent, colorimetric, and affinity-based, that can serve as HTP surrogates for electrochemical function during biosensor discovery and optimization.

Taken together, SELEX and FACS-based screening approaches illustrate how nonelectrochemical assays can perform large library screening essential for electrochemical biosensor design. Both methods enable the interrogation of extraordinarily large variant libraries, producing sequence-resolved data sets. This breadth of information can provide a foundation for identifying structure–function relationships, extracting design rules and training predictive models that relate sequence and activity to sensor performance. Importantly, the outputs of these assays (aptamers and enzymes) are directly transferable to electrode-based platforms, where they can be evaluated under electrochemical operating conditions. By enabling large-scale discovery and optimization upstream of electrochemical validation, SELEX and FACs can transform electrochemical biosensor development from a low-throughput trial and error process into a data-rich, iterative design workflow with the potential for substantially quicker and improved sensor development.

#### 1.2.3 Opportunities in Directed Evolution

With scalable surrogate screening platforms becoming available as described above, directed evolution should become a particularly powerful strategy for electrochemical biosensor development. In catalytic electrochemical sensors, signal output is directly proportional to electron flux, which in turn depends on enzyme turnover, substrate binding, and electron-transfer efficiency.^83,84^ Directed evolution offers a systematic route to optimize each of these parameters simultaneously, rather than tuning them independently through rational design. Furthermore, because sensing often occurs in complex environments with numerous potential interferents, the ability to evolve enzymes under selection pressures that penalize cross-reactivity would be an advantage over purely rational approaches.^65,66^

Redox enzymes are promising targets for evolution-enabled electrochemical sensing. A large fraction of directed evolution efforts to date have focused on oxidoreductases, including dehydrogenases, oxidases, and cytochrome p450 monooxygenases, many of which utilize electrons to drive substrate transformations.^85,86^ Cytochrome p450s, in particular, have been extensively evolved for altered substrate scope, improved coupling efficiency, and enhanced regio- and stereoselecti­vity.^87,88^ Notably, P450s have already been interfaced with electrodes to facilitate both direct electron transfer (DET) and mediated electron transfer (MET), demonstrating that electrode-driven catalysis is feasible. In such systems, electron flow from the electrode to the enzyme can be linked to substrate turnover, suggesting a straightforward route to catalytic electrochemical sensing: substrate-dependent electron consumption can be directly measured as a current response.^89–92^

From a biosensing perspective, the enzyme-directed evolution literature on P450s and related redox enzymes represents a largely untapped resource. Many prior evolution campaigns have already optimized the properties most relevant to biosensor performance, including substrate specificity, catalytic efficiency, and resistance to off–target reactions.^88,89^ By repurposing these enzymes, or evolving them explicitly under sensor-relevant selection pressures, it should be possible to construct a catalytic biosensor in which electron flux serves as a quantitative proxy for analyte concentration.

Beyond sensor design, optical surrogate assays for directed evolution can also be applied to the engineering of multiple systems relevant to electrochemical catalysis. Fluorescence or colorimetric readouts have been employed to screen large enzyme libraries for enhanced product yield, facilitating the identification of variants that were subsequently validated electrochemically.^93–95^ These examples highlight how nonelectrochemical screening can guide the evolutionary optimization of catalytic architectures prior to electrochemical validation. Looking forward, the integration of directed evolution with data-driven modeling and automation presents an opportunity to reshape electrochemical biosensor development. Large-scale mutational data sets generated during evolution campaigns can be used to identify sequence features that control electron transfer, substrate discrimination, and catalytic efficiency, enabling more informed design in subsequent rounds. When combined with HTP screening, machine learning-guided library design, and automated experimentation, directed evolution could enable rapid development of electrochemical biosensors.

#### 1.2.4 Opportunities in Building Electrochemical Sensors by De Novo Design of Enzymes

Recent advances in de novo protein design have demonstrated that it is now possible to create entirely new sensing and catalytic functions from first principles.^96–98^ In these systems, rationally designed binding domains are coupled to optical reporters, enabling real-time visualization of analytes in living cells.^99–101^ However, as discussed above, such optical approaches are often poorly suited for applications outside of laboratory settings. Notably, despite major advances in de novo protein design, there are still no examples of fully de novo–designed proteins that demonstrate enzyme-like, electron-coupled redox catalysis or are explicitly engineered for electrochemical sensing.

However, several successful results from de novo protein design are directly translatable to electrochemical readouts. One strategy involves analyte-induced assembly of designed protein complexes, such as split enzymes with an attached split luciferase, in which binding of a target molecule drives reconstitution of an active reporter (Figure 8A).^99,102^ Analogous architectures could be built for electrochemical sensing, in which analyte-induced association brings an electrode-anchored protein into proximity with a second designed protein bearing an appended electroactive small molecule, such as methylene blue or other redox reporters (Figure 8B). In this configuration, molecular recognition would be transduced into a measurable electrochemical signal through distance-dependent electron transfer, mirroring the logic of structure-switching aptamer-based electrochemical sensors.

**Figure 8:**
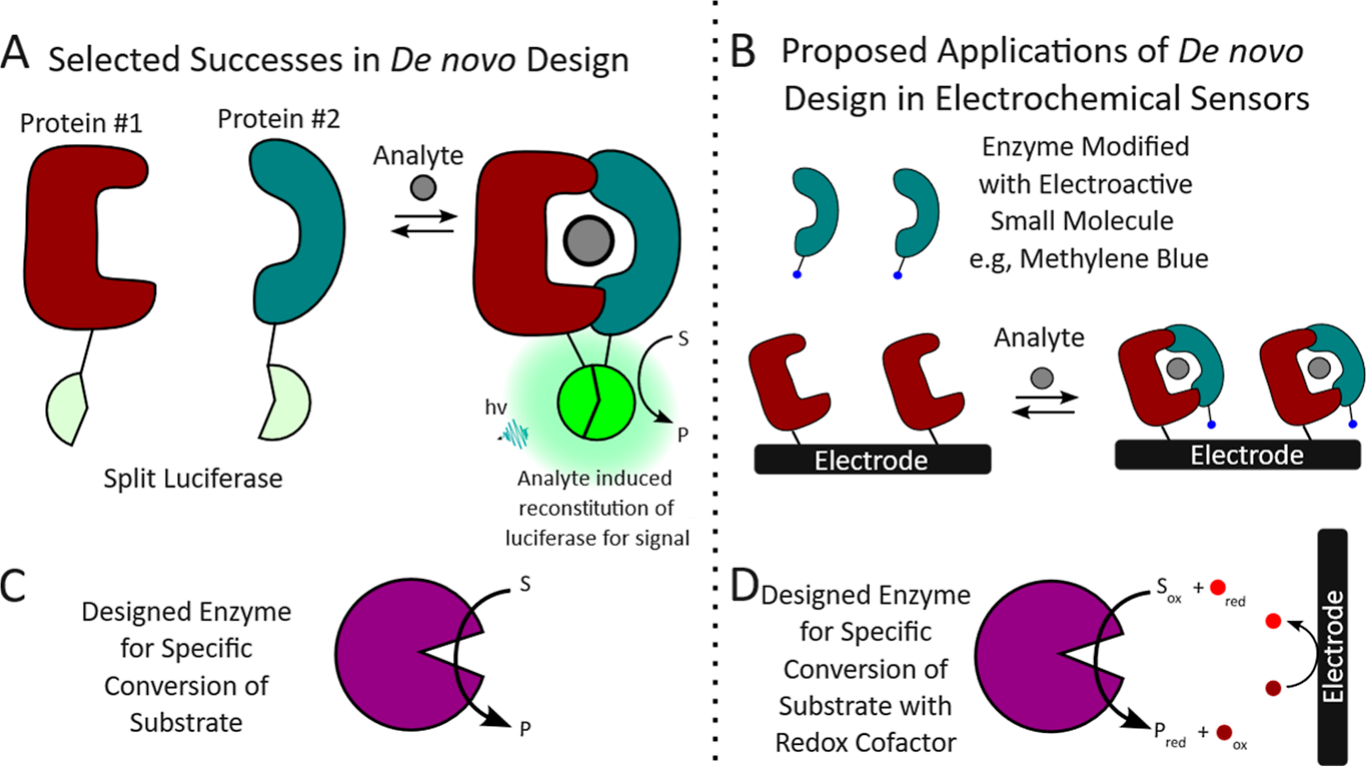
(A,C) Current application of de novo design in building novel proteins (B,D) extension of the current state of de novo design toward building electrochemical sensors.

Aside from simple molecular recognition, de novo protein design has also produced a growing number of enzymes capable of catalyzing specific chemical transformations, typically through stabilization of well-defined transition states and precisely organized active sites. To date, most such efforts have focused on bond-forming isomerization, or hydrolytic reactions, rather than redox chemistry (Figure 8C).^97,103,104^ Extending these principles to the design of redox enzymes would be a natural next step. In particular, de novo enzymes that catalyze oxidation–reduction reactions using cofactors that can be regenerated at an electrode would enable catalytic electrochemical sensing, in which substrate turnover is directly coupled to electron flow. In such systems, the rate of cofactor oxidation or reduction would serve as a quantitative proxy for analyte concentration (Figure 8D).^104^ Taken together, recent advances in de novo protein design, particularly the ability to precisely arrange functional residues, engineer tight molecular recognition, and catalyze specific chemical reactions, represent an untapped opportunity to expand electrochemical biosensing beyond the constraints imposed by naturally evolved proteins.

## 2 Conclusions

Electrochemical biosensors are poised to play an increasingly central role in wearable diagnostics, point-of-care testing, and continuous monitoring due to their inherent compatibility with miniaturized electronics, low power requirements, and proven clinical relevance. However, despite decades of progress in materials science and device engineering, the development of new electrochemical biosensors remains fundamentally constrained by the low throughput of electrochemical screening methods. This limitation has hindered systematic exploration of recognition elements, catalytic components, and signal-transduction architectures, resulting in sensor development pipelines that rely heavily on incremental, trial-and-error optimization.

Throughout this perspective, we have highlighted how nonelectrochemical HTP surrogate assays, drawn from synthetic biology, chemical biology, and protein engineering, offer a path to overcoming this bottleneck. Approaches such as SELEX for aptamer discovery, fluorescence and FACS-based enzyme screening, directed evolution, and de novo protein design enable exploration of molecular and sequence space on scales that are currently inaccessible to electrochemical measurements alone. By decoupling large-scale discovery and optimization from electrochemical readout, these platforms will allow electrochemistry to be repositioned as a validation and deployment layer, rather than a discovery bottleneck.

Looking forward, the integration of surrogate screening assays, which can be incredibly data rich, will allow for data-driven modeling and machine-learning guided library design transforming electrochemical biosensor development into a scalable, predictive and iterative design process. Rather than adapting naturally evolved recognition elements to electrochemical formats post hoc, future biosensors can be purpose-built and evolved using tools that explicitly optimize for selectivity, robustness, and electron-transfer efficiency under sensor-relevant conditions. Continued development of HTP screening techniques that serve as faithful proxies for electrochemical measurements will be critical to realizing this vision, enabling functional evolution and optimization at scales inaccessible to electrochemical readouts alone. This shift will require closer integration between electrochemistry and the methodologies of chemical and synthetic biology, leading to faster development cycles, improved sensor performance, and broader access to electrochemical biosensing technologies.
